# Contribution of the RgfD Quorum Sensing Peptide to *rgf* Regulation and Host Cell Association in Group B *Streptococcus*

**DOI:** 10.3390/genes8010023

**Published:** 2017-01-06

**Authors:** Robert E. Parker, David Knupp, Rim Al Safadi, Agnѐs Rosenau, Shannon D. Manning

**Affiliations:** 1 Department of Microbiology and Molecular Genetics, Michigan State University, East Lansing, MI 48824, USA; parke274@msu.edu (R.E.P.); knuppdav@msu.edu (D.K.); rimalsafadi@gmail.com (R.A.S.); 2 Infectiologie et Santé Publique ISP, Institut National de la Recherche Agronomique, Université de Tours, Equipe Bactéries et Risque Materno-fœtal, UMR1282 Tours, France; rosenau@univ-tours.fr

**Keywords:** *Streptococcus agalactiae*, quorum sensing, colonization, group B *Streptococcus*

## Abstract

*Streptococcus agalactiae* (group B *Streptococcus*; GBS) is a common inhabitant of the genitourinary and/or gastrointestinal tract in up to 40% of healthy adults; however, this opportunistic pathogen is able to breach restrictive host barriers to cause disease and persist in harsh and changing conditions. This study sought to identify a role for quorum sensing, a form of cell to cell communication, in the regulation of the fibrinogen-binding (*rgfBDAC*) two-component system and the ability to associate with decidualized endometrial cells in vitro. To do this, we created a deletion in *rgfD*, which encodes the putative autoinducing peptide, in a GBS strain belonging to multilocus sequence type (ST)-17 and made comparisons to the wild type. Sequence variation in the *rgf* operon was detected in 40 clinical strains and a non-synonymous single nucleotide polymorphism was detected in *rgfD* in all of the ST-17 genomes that resulted in a truncation. Using qPCR, expression of *rgf* operon genes was significantly decreased in the ST-17 *ΔrgfD* mutant during exponential growth with the biggest difference (3.3-fold) occurring at higher cell densities. Association with decidualized endometrial cells was decreased 1.3-fold in the mutant relative to the wild type and *rgfC* expression was reduced 22-fold in *ΔrgfD* following exposure to the endometrial cells. Collectively, these data suggest that this putative quorum sensing molecule is important for attachment to human tissues and demonstrate a role for RgfD in GBS pathogenesis through regulation of *rgfC*.

## 1. Introduction

*Streptococcus agalactiae*, or group B *Streptococcus* (GBS), resides as a commensal in the gastrointestinal and/or urogenital tracts in up to 40% of healthy men and women but is an opportunistic pathogen presenting a threat to newborns, pregnant women, the chronically ill, and the elderly [1]. In neonates, GBS is a leading cause of meningitis and sepsis. Although there has been a reduction in the incidence of neonatal early onset disease (EOD) over the past 30 years [2], GBS is still a major concern in both industrialized and developing nations, and there remain significant gaps in our understanding of the molecular mechanisms of pathogenesis. The identification of features that allow one GBS strain to become more invasive than another is incomplete. Several studies utilizing multilocus sequence typing (MLST), a method targeting seven conserved housekeeping genes [3], have shown that most strains belong to one of four clonal complexes (CCs): 1, 17, 19, and 23. Strains belonging to CC-17, however, have been shown to cause an increased frequency of neonatal infections [3,4] and were suggested to be more virulent with unique features that impact disease development and progression [5,6,7,8].

While GBS is well adapted to survival in the host, crossing restrictive barriers like the extraplacental membranes and blood-brain barrier presents a challenge to the bacterium as disease progression requires the complex regulation of multiple virulence factors [9,10]. The ability to respond to environmental cues occurs through transcriptome remodeling, which facilitates adaptation and survival in distinct niches [11]. Indeed, remodeling of the GBS transcriptome has been observed in response to growth temperature and exposure to other host-specific environments [12,13,14]. For most bacterial pathogens, the ability to recognize extracellular stimuli and respond occurs via signal transduction systems (STS), with the most common being two-component systems (TCSs) [15]. Most TCSs are composed of a membrane-bound sensor kinase, which reacts to an extracellular stimulus by phosphorylating and activating a response regulator that serves as a transcription factor driving downstream behavioral changes [16]. The number of TCSs in bacterial chromosomes have been shown to correlate with the genome size at a rate of ~2.3 TCSs per 1 Mb for genomes up to 5 Mb [17]. GBS has a disproportionately high number of TCSs with 17–20 predicted for the 2.2 Mb genome [18]. Several of these systems have been shown to play a role in pathogenesis, including the controller of virulence (CovR/S) [19], the regulator of D-Alanyl-lipotechoic acid biosynthesis (DltR/S) [20], the competence and β-lactam-resistance promoting system (CiaR/C) [21], and the regulator of fibrinogen binding (RgfA/C) [22].

The Rgf system, encoded by the *rgfBDAC* operon, was identified as a polycistronically transcribed system that promotes binding of host cell components through the regulation of cell surface proteins including the fibronectin binding protein, *scpB*, and two fibrinogen binding proteins, *fbsA* and *fbsB* [22,23]. Importantly, the *rgf* operon is homologous to the accessory gene regulator operon (*agrBDCA*), a TCS found in staphylococci [22]. The *agr* TCS is a well-studied quorum sensing circuit important for virulence via the regulation of secreted virulence factors and surface proteins [24,25]. Regulation of this operon, however, is complex and has been linked to multiple factors [24,25,26,27]. Similar to the *agr* system, the *rgf* operon is composed of a putative ABC transporter, *rgfB*, a putative quorum sensing protein, *rgfD*, and the TCS *rgfA/C* [22].

Genetic variation has been described in both the *S. aureus agr* and GBS *rgf* system. Mutations conferring a non-hemolytic, non-invasive phenotype have been detected in the *agr* operon from strains recovered from patients [24,28]. For the *rgf* operon, one study identified a truncation in the gene encoding the response regulator, *rgfA*, in several clinical strains and deletion of both *rgfC*, the sensor histidine kinase, and *rgfA* resulted in increased virulence in a mouse and rat model [29]. This increase was possibly due to other virulence mechanisms including increased sialic acid production and capsule operon transcription, which were both altered in the deletion mutant. The same study also found that extrachromosomal *rgfC* expression altered the transcriptome, indicating that regulation of the sensor histidine kinase may be important for GBS pathogenesis [29]. Separate analyses of the genome of NEM316, a serotype III ST-23 GBS strain isolated from a fatal case of neonatal septicemia, also identified a large deletion within *rgfD*, which encodes a putative auto-inducing peptide, and part of *rgfC* [30]. The role of *rgfD* and different *rgfD* mutations on the regulation of the *rgf* operon in GBS, however, has not been examined nor has the impact of both on phenotypes relevant for pathogenesis. We therefore sought to investigate the contribution of *rgfD* to biofilm production, host cell association, and operon regulation in distinct growth stages and following exposure to decidualized endometrial cells.

## 2. Materials and Methods

### 2.1. Bacterial Strains, Growth Conditions, and rgf Sequence Analysis

GBS was cultured in Todd-Hewitt broth (THB) or agar (THA) or trypticase soy agar plus 5% sheep’s blood (Becton Dickinson, Franklin Lakes, NJ, USA) at 37 °C with 5% CO_2_. Growth curves were performed in THB using the same conditions with samples taken for determination of OD_595_ at different times. Three serotype III, CC-17 strains (GB00451, GB00546, and GB00097) were used to quantify *rgf* transcription by growth phase. Mutagenesis was performed in GB00451 (ST-17) and GB00012 (ST-1).

To examine sequence variation, additional *rgf* operon sequences were extracted from 40 draft genomes sequenced by the J. Craig Venter Institute (Table 1) using the Basic Local Alignment Search Tool (BLAST) available in the National Center for Biotechnology Information (NCBI) with strain O90R as the *rgf* reference sequence (AF390107.1) [22]. All base locations in the 40 genomes are named relative to the 3320 bp O90R sequence, which begins 94 bp prior to the start of *rgfB* in the *rgf* operon. Multiple alignments were performed using the ClustalW algorithm in MegAlign and a Neighbor joining phylogeny based on p-distance was generated using MEGA6 with bootstrapping [31]. The 40 clinical strains, which were recovered from colonized mothers or young adults, were previously characterized by MLST [7,32]. Although biofilm production was performed previously using OD_595_ values ≥1.8 as the cutoff for strong biofilms [33], this study sought to examine the relationship between biofilm level and *rgf* sequence variation, which was not examined initially.

### 2.2. rgfD Mutagenesis and Complementation

Mutagenesis was performed using a double-homologous recombination strategy with the pG+host5 thermosensitive plasmid [34] for the deletion of *rgfD* as described [35]. Flanking regions were amplified by PCR using primers rgfD_del 1 and 2 and rgfD_del 3 and 4 (Table 2). An assembly PCR resulting in a single product was accomplished using equal amounts of the flanking products with the primers rgfD_del1 and rgfD_del4. Restriction digestion using *BamHI* and *KpnI* (New England Biolabs, Ipswich, MA, USA) of the resulting product and the plasmid pG+Host5 were performed followed by ligation and electroporation into Max Efficiency DH5α *Escherichia coli* electrocompetent cells (Thermo Fisher Scientific, Waltham, MA, USA) using a Micropulser (Bio-Rad, Hercules, CA, USA). The plasmid was confirmed to be present by PCR amplification with primers PGhost 4630 and PGhost 5117 and sequencing of the resulting product followed by electroporation into GB00451 and growth at 28 °C with erythromycin (2 µg/mL). Chromosomal integration of pG+host:*ΔrgfD* was selected for by growth on agar at 40 °C in the presence of erythromycin. Excision and loss of the plasmid was stimulated by growth at 28 °C without antibiotics in broth for six generations followed by dilution and plating. Single colonies were tested for erythromycin susceptibility to ensure plasmid loss and PCR was performed using primers rgfD_del 5 and 6 to identify a mutant with a gene deletion (GB00451*ΔrgfD*). Complementation of *rgfD* was completed using the pLZ12 plasmid with a constitutive *rofA* promoter sequence regulating transcription [36]. For construction, *rgfD* was amplified from GB00012 with Plz:rgfD F and R, digested with *Pst*I and *BamH*I enzymes, and ligated into the pLZ12 plasmid. The constructed plasmid was transformed into the DH5α MAX Efficiency Chemically-Competent Cells by Invitrogen™ (Thermo Fisher Scientific, Waltham, MA, USA) and chloramphenicol resistant transformants were identified. The plasmid was extracted and electroporated into GB00451*ΔrgfD* competent cells, and transformants were selected for growth on THA and chloramphenicol (3 µg/mL).

### 2.3. Association Assays

Telomerase-immortalized human endometrial stromal cells (T-HESCs) were decidualized and grown to approximately 50% confluence followed by treatment with 0.5 mM 8-bromo-cyclic adenosine monophosphate (Sigma-Aldrich, St. Louis, MO, USA) for 3–6 days as described [37]. Decidualization was confirmed by examining the expression of prolactin and insulin-like growth factor-binding protein 1. Assays were performed in triplicate at least three times when cells reached 100% confluence. GBS was washed with phosphate-buffered saline (PBS) and resuspended in infection medium (HESC medium with 2% charcoal-treated fetal bovine serum, insulin, human transferrin, and selenous acid without antibiotics) following overnight growth in THB. Host cells were washed three times with PBS and infected with GBS at a multiplicity of infection (MOI) of one bacterial cell per host cell. After 2 h at 37 °C with 5% CO_2_, samples were taken, diluted, and plated to quantify bacteria (CFU/mL). Each well was washed three times with PBS to remove non-adherent bacteria, and host cells were lysed with 0.1% Triton X-100 (Sigma-Aldrich) for 30 min at 37 °C and mixed to liberate intracellular bacteria. After serial dilution, lysates were plated on THA, incubated overnight at 37 °C, and quantified (CFU/mL). All data were expressed as percentages of the total number of bacteria per well after 2 h.

### 2.4. RNA Extraction, Preparation, and Quantitation

RNA was extracted, cDNA was synthesized and transcripts were quantified as previously described [37]. For collection, samples were added to two volumes of RNA Protect (Qiagen, Germantown, MD, USA) and pelleted followed by RNA extraction using the RNeasy Kit (Qiagen). DNA was removed with TURBO™ DNase (Thermo Fisher Scientific) and purified RNA was quantified. For samples exposed to host cells, total RNA was precipitated following Turbo DNase treatment and bacterial RNA was separated using the MICROB*Enrich™* Kit by Ambion (Thermo Fisher Scientific). Following purification, 1 µg of RNA was used for reverse-transcription with the iScript Reverse Transcription Kit (Bio-Rad), while the iQ SYBR Supermix (Bio-Rad) was used for quantitative RT-PCR (qRT-PCR) in 15 µL reactions with 10 μM (each) of gene-specific primers (Table 2). Products were amplified and quantified using a CFX384 Touch™ Real-Time PCR detection system (Bio-Rad) under the following conditions: 1 cycle of 3 min at 95 °C and 39 cycles of 95 °C for 10 s and 60 °C for 30 s. Relative transcript quantities were calculated using the comparative threshold cycle (*C_T_*) method (2^−Δ*CT*^) [38] with *gyrA* as the internal control gene.

### 2.5. Statistical Analysis

Data shown were either pooled from or were representative of at least three independent experiments performed in triplicate. The *t*-test was used to compare differences in expression levels across groups of strains, while the paired ratio *t*-test was used to compare percent association to host cells. The likelihood Chi-square test was used to examine differences in categorical variables. Analyses were performed in GraphPad Prism (version 6.0; GraphPad Software, Inc., La Jolla, CA, USA) and Epi Info™ (CDC, Atlanta, GA, USA). *p* ≤ 0.05 was considered significant.

## 3. Results

### 3.1. Allelic Variation in rgf among Diverse GBS Lineages

**Figure 1:**
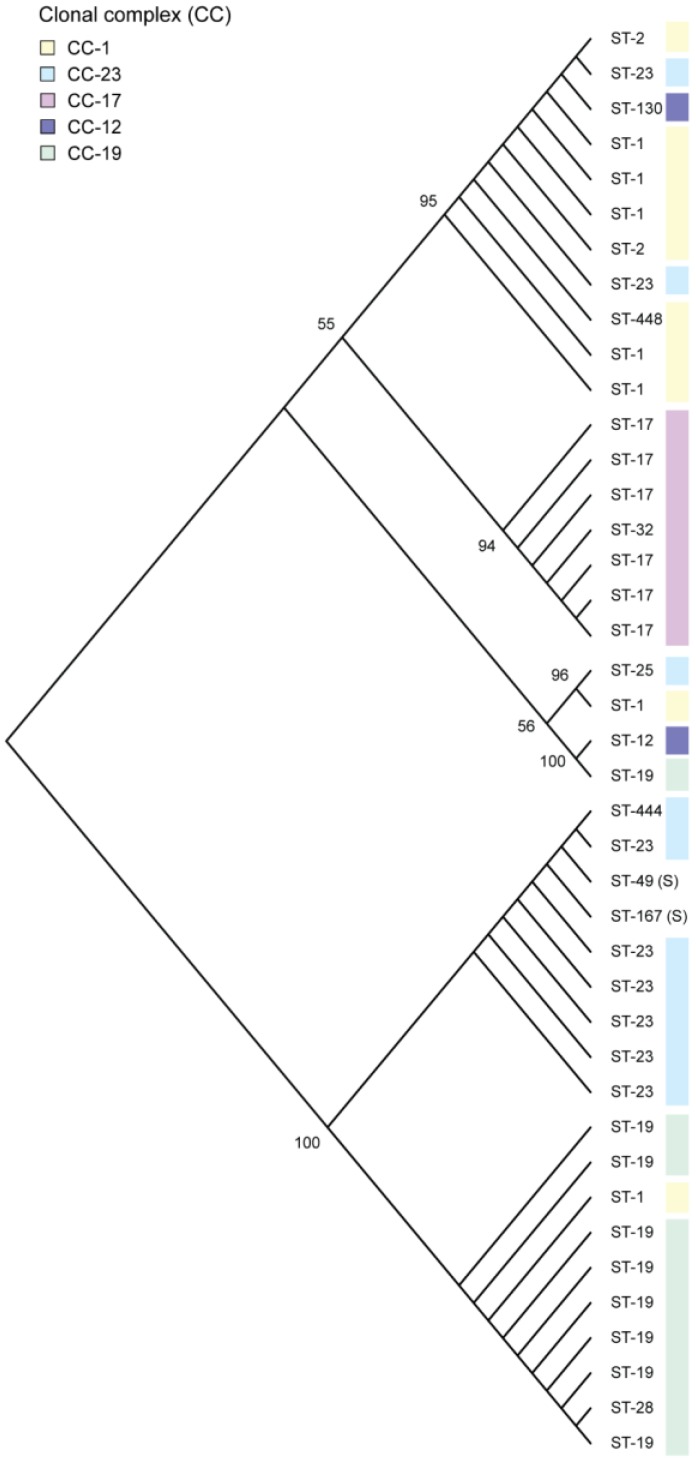
Neighbor joining phylogeny of *rgf* operon alleles by multilocus sequence type (ST). The evolutionary distances between *rgf* operon sequences (3320 bp) for 41 strains of different STs were calculated using the p-distance method, which is represented as the number of base differences per site. The bootstrap test (1000 replicates) values are represented at the nodes. The *rgfC* sequence, which was classified as complete or with an 881 bp deletion, contributed to the clustering observed in the phylogeny. S = singleton.

Because sequence variation within the *rgf* operon has been observed [23,39], we compared the O90R *rgf* reference sequence [22] to 40 *rgf* sequences from clinical strains representing 14 STs. In all, 39 strains were classified as belonging to five CCs including CC-1 (*n* = 10), CC-12 (*n* = 2), CC-17 (*n* = 7), CC-19 (*n* = 10), and CC-23 (*n* = 10); two strains were singletons. Phylogenetic analysis of the complete 3320 bp *rgf* operon extracted from NCBI resulted in two *rgf* clusters (Figure 1), which differed based on the presence of an 881 bp deletion within *rgfC* at position 2328 as well as multiple single nucleotide polymorphisms (SNPs) within both *rgfA* and *rgfC*. A total of 21 (52.5%) strains contained the complete *rgf* operon with an intact *rgfC*, while the remaining 19 (47.5%) strains contained the 881 bp *rgfC* deletion.

When stratified by ST, strains of the same ST were more likely to cluster together. The 20 ST-19 and ST-23 strains, for example, clustered together on the tree depending on whether they harbored the complete (*n* = 4) or deleted (*n* = 16) version of *rgfC*. The same was true for CC-1 strains, though one ST-1 strain had the *rgfC* deletion and clustered separately from the others. By contrast, strains belonging to ST-17 were homogeneous with only three detectable SNPs among all seven ST-17 genomes. These SNPs were located within *rgfD* (T1115G), *rgfA* (A1848T), and *rgfC* (G2338A) and each mutation was exclusive to one of the three different ST-17 strains. Relative to the other STs, two unique non-synonymous SNPs were detected in all of the ST-17 strains. The first SNP (C246A) is located in *rgfB* and the second (A1131T) is located 54 bp into *rgfD*. Importantly, the *rgfD* SNP results in a truncated coding sequence after 17 amino acids due to the introduction of a stop codon. This finding suggests that *rgfD* may function differently in ST-17 strains relative to strains belonging to other lineages. Mutations within *rgfD* also resulted in the separate clustering of a ST-12 and ST-19 strain with a complete *rgfC* near the bottom of the top branch of the phylogeny. Both strains had three unique SNPs, G1044A, C1048T, and A1054G, located 21 bp, 25 bp, and 31 bp into *rgfD*, respectively, as well as an additional SNP (A3018T) in *rgfC* that was shared only with the seven ST-17 strains. Only C1048T and A1054G in *rgfD* represent non-synonymous mutations.

### 3.2. Association between rgf Variation and Biofilm Production

Since allelic variation in the *agr* system has previously been related to biofilm production in *S. aureus* [40], we assessed the importance of *rgf* allelic variation on biofilm phenotypes. Of the 40 clinical strains examined, 13 (32.5%) were previously classified as strong biofilm producers and 27 (67.5%) were weak. Those strains possessing a complete *rgfC* were not more likely to produce a strong biofilm relative to the strains containing the *rgfC* deletion (*p* = 0.83). Among the 21 strains with a complete *rgfC*, 28.6% (*n* = 6) were strong biofilm producers relative to 36.8% (*n* = 7) of strains with the *rgfC* deletion. It is notable that all but one of the seven CC-17 strains containing the *rgfD* truncation were classified as weak biofilm producers. Although the strains containing a complete *rgfD* were 3.4 times more likely to form a strong biofilm, the association was not statistically significant (95% confidence interval: 0.42, 85.59; Fisher’s exact *p* = 0.39), which may be due to the small sample size.

### 3.3. rgfD-Dependent Expression of the rgf Operon

**Figure 2:**
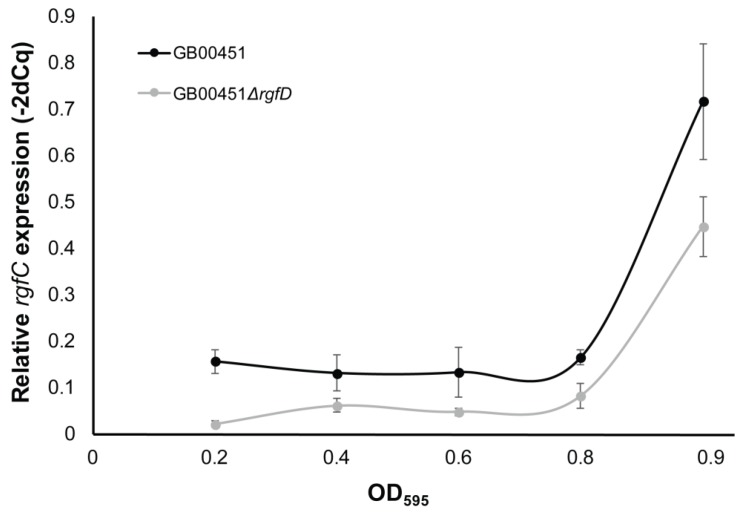
Expression of *rgfC* increased over the growth phase in sequence type (ST)-17 strains. *rgfC* expression was assessed in three clinical ST-17 strains including GB00451 (shown here), which was compared to GB00451*ΔrgfD*. The relative *rgfC* transcript quantity is represented as the optical density (OD)_595_ increases. Error bars represent the standard deviation between the strains at a given OD_595_.

**Figure 3:**
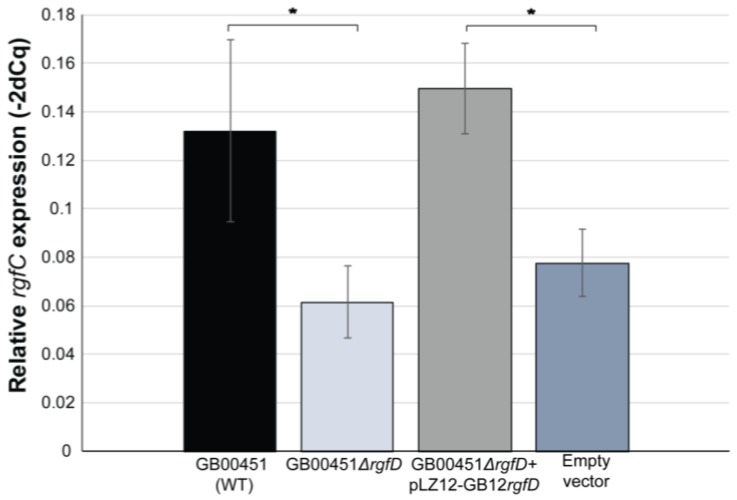
Expression of *rgfD* is necessary for *rgfC* expression. Comparison of the relative *rgfC* transcript quantity between the GB00451 wild-type (WT) and GB00451*∆rgfD* mutant during early mid-log (OD_595_ = 0.4) growth. The GB00451*∆rgfD* mutant complemented with GB0012*rgfD* on the pLZ12 plasmid (pLZ12-GB12*rgfD*) and complementation with pLZ12 alone (empty vector) are also shown. Bars represent the standard deviation of four biological replicates. * *t*-test *p*-value < 0.05.

Since quorum-sensing controlled systems are characterized by increased expression when the extracellular inducer reaches a specific concentration, we sought to quantify *rgf* expression in a subset of GBS strains. The *rgf* operon was previously shown to be transcribed polycistronically [22]; therefore, we examined expression of *rgfC*, the gene encoding the sensor histidine kinase (*rgfC*), in three ST-17 clinical strains over time. No difference in relative *rgfC* transcript quantity was observed between the three clinical strains at any of the time points. It is important to note that all three strains contained complete *rgf* operons with a complete *rgfC* and genetically identical *rgfB* and *rgfD* genes. Next, we deleted *rgfD* in one of the three CC-17 strains, GB00451 (wild type; WT), in order to compare *rgfC* expression along the growth curve to the same strain lacking *rgfD*. Samples were also subcultured to ensure that the bacterial densities were similar at each OD_595_ value; no difference in colony forming units (CFU) was observed between the WT and GB00451*ΔrgfD* mutant (data not shown). Significantly reduced relative *rgfC* transcript quantity was observed at all growth points in the GB00451*ΔrgfD* mutant relative to the WT (Figure 2). The largest difference was observed in lag phase (OD_595_ = ~0.2) with relative expression values of 0.16 ± 0.03 and 0.02 ± 0.01 for WT and mutant, respectively. In early log phase (OD_595_ = ~0.4), *rgfC* expression was reduced from 0.13 ± 0.04 to 0.06 ± 0.01 in the WT versus mutant (*p* = 0.04) and at mid-log phase (OD_595_ = ~0.6), expression values of 0.13 ± 0.05 and 0.05 ± 0.008 were observed for the WT and mutant (*p* = 0.05). At late log phase, the WT had a significantly higher level of *rgfC* expression (0.17 ± 0.02) compared to the GB00451*ΔrgfD* mutant (0.08 ± 0.03; *p* = 0.01). Although expression of *rgfC* was highest for both strains at stationary phase, the level of expression in the mutant (0.44 ± 0.06) was still significantly lower than in the WT (0.72 ± 0.12; *p* = 0.03). Complementation of GB00451*ΔrgfD* with the pLZ12 plasmid containing the truncated version of *rgfD* from GB00451 (WT) was not capable of restoring *rgfC* expression. To determine whether this result was partly due to the *rgfD* mutation in the WT strain, complementation with pLZ12-*rgfD* from GB00012*,* a ST-1 strain lacking the *rgfD* truncation*,* was performed. Importantly, complementation of GB00451*ΔrgfD* with pLZ12-*rgfD* from GB00012 (Figure 3) was sufficient to restore relative expression of *rgfC* to 0.15 ± 0.02 at OD_595_ = 0.4 compared to the empty vector control (0.08 ± 0.1); *t*-test *p* < 0.01).

To determine whether RgfD alters expression of other genes that were suggested to be regulated by the *rgf* operon, the WT and GB00411*ΔrgfD* mutant were examined for expression changes in the gene encoding the fibrinogen binding surface protein (*fbsB*), which is activated by *rgfA/C* [23]. Notably, relative *fbsB* transcript quantity was similar for the WT (0.010 ± 0.003) and GB00451*ΔrgfD* (0.008 ± 0.003) mutant at OD_595_ = 0.4 as well as OD_595_ = 0.6 (0.012 ± 0.005 for the WT versus 0.012 ± 0.006 for GB00451*ΔrgfD*). Expression levels in stationary phase (OD_595_ = 0.8) were slightly more variable, though there was still no significant difference between the WT (0.0007 ± 0.0004) and mutant (0.0009 ± 0.0008).

### 3.4. Role of rgfD in Association with Host Cells and Biofilm Production

**Figure 4:**
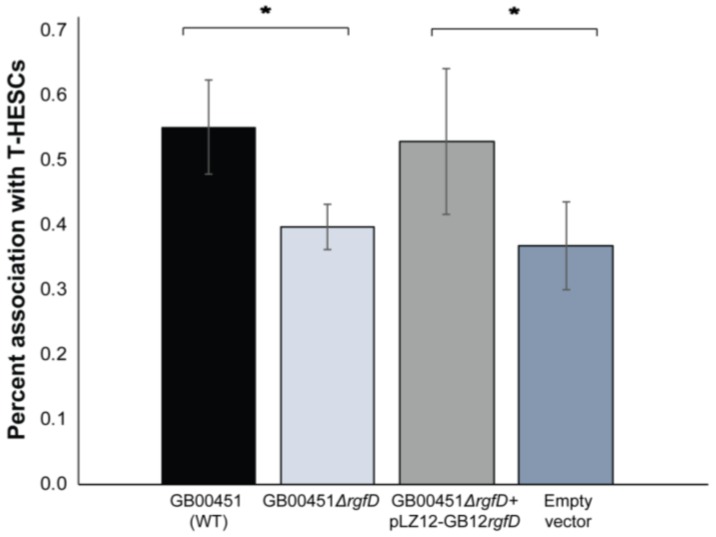
*rgfD* plays a role in association with decidualized endometrial stromal cells. Association percentages for GB00451 (WT) and GB00451*ΔrgfD* with telomerase-immortalized human endometrial stromal cells (T-HESCs) are shown as well as the percentages for GB00451*ΔrgfD* complemented with pLZ12 containing *rgfD* from GB00012 (pLZ12-GB12*rgfD*) and the empty vector (pLZ12 only). The histogram represents a single biological replicate with three technical replicates and error bars representing the standard deviation between technical replicates; the assay was performed four times in triplicate with identical trends per assay. * paired ratio *t*-test *p*-value < 0.05.

Since the *rgf* operon has been shown to promote binding to host cell components like fibrinogen [23], the ability to associate with T-HESCs was investigated. Interestingly, the GB00451*ΔrgfD* mutant had an average 1.3-fold decrease in the ability to associate with the decidualized endometrial cells compared to the WT. Association with T-HESCs was 0.40% ± 0.03% for GB00451*ΔrgfD* compared to 0.55% ± 0.07% for the WT (ratio *t*-test *p* < 0.03) (Figure 4). The empty vector control had an average 1.6-fold reduction in the level of association with T-HESCs compared to GB00451*ΔrgfD* complemented with pLZ12 containing *rgfD* from GB00012. The association level for the complemented mutant was 0.53% ± 0.11% versus 0.37% ± 0.07% for the empty vector (ratio *t*-test *p* = 0.002). It is important to note that even though the trend remained consistent across biological replicates, association percentages varied between experiments. When biofilms were examined, no difference in biofilm production was observed between the WT, GB00451*ΔrgfD* mutant, both complemented mutants, or empty vector control.

**Figure 5:**
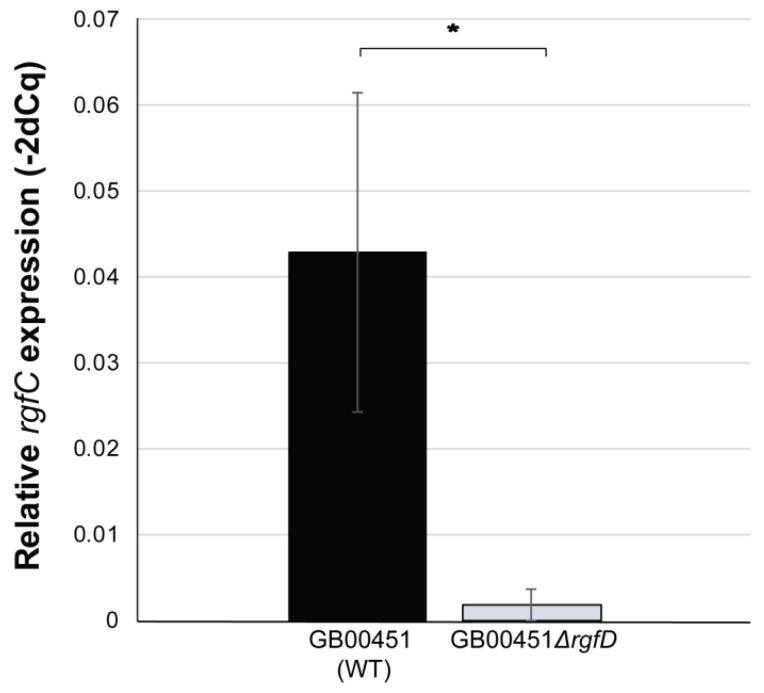
*rgfC* is upregulated by *rgfD* following exposure to decidualized endometrial stromal cells. Comparison of the relative *rgfC* transcript quantity between the GB00451*rgfD* wild-type (WT) and GB00451*∆rgfD* mutant following 2 h exposure to telomerase-immortalized human endometrial stromal cells (T-HESCs). Bars represent the standard deviation of three biological replicates. * *t*-test *p*-value < 0.05.

Because the association assays were performed in different conditions than the *rgfC* expression analysis, we also sought to compare *rgfC* expression in the WT, *rgfD* mutant, complemented *rgfD* mutant, and empty vector control to determine whether differential regulation of the operon was detectable following host cell exposure. Notably, a 22.8-fold reduction in *rgfC* expression was observed in the GB00451*ΔrgfD* mutant compared to the WT following a 2 h exposure to decidualized T-HESCs; relative transcript levels were 0.0019 ± 0014 and 0.043 ± 0.019, respectively (Figure 5). No difference in *rgfC* expression was observed, however, between the complemented and empty vector controls with relative transcription values of 0.029 ± 0.01 and 0.031 ± 0.01, respectively, following exposure to T-HESCs.

## 4. Discussion

Because the *rgf* operon was found to facilitate binding to host cell components and impact virulence in vivo, [22,23,29] we sought to better understand the role of the putative autoinducing peptide, RgfD, in phenotypes important for colonization. Similar to prior studies [29,39], we have demonstrated *rgf*-dependent expression of the *rgf* operon and have identified genetic variation in the *rgf* operon genes among a diverse set of GBS strains. The large 881 bp deletion within *rgfC* is notable given that it was present in almost half of the 40 strains recovered from women with asymptomatic GBS colonization. Since these strains represented multiple STs and were collected from patient populations in different geographic locations and time periods, the presence of this mutation suggests parallel evolution in the *rgfC* locus. Evidence for gene loss as well as lateral transfer and gene duplication have been described for genes involved in other quorum sensing systems (e.g., *Pseudomonas* [41]).

The identification of a point mutation within *rgfD* that was exclusive to the seven clinical strains belonging to CC-17, the lineage most commonly associated with neonatal disease, [4] is also noteworthy. The A1131T mutation results in transcription of a premature stop codon within the *rgfD* open reading frame to encode a truncated protein. Because of this mutation, it is possible that *rgfD* functions differently in ST-17 strains versus strains of other lineages with a complete *rgfD*. In *S. aureus*, sequence variation has been observed within the auto-inducing peptide gene *(agrD*) and was shown to influence activation of the two component system [42,43]. The AgrD peptide was also found to be post-translationally modified and reduced to a functional eight amino acid peptide [44]. In all phases of growth, we found that the truncated *∆rgfD* mutant had decreased expression of *rgfC*, which encodes the sensor histidine kinase and represents the last of the four genes in the polycistronically transcribed operon [22]. These data suggest that this truncated RgfD protein is functional in this CC-17 strain and likely serves as an auto-inducing peptide in conjunction with other factors. This hypothesis is in agreement with *agr* regulation in *S. aureus* in which there are several factors affecting expression besides AgrD [26,27]. Furthermore, maximum *rgfC* expression was observed as the cells entered stationary phase in both the WT as well as the GB00451*∆rgfD* mutant, suggesting that other factors can impact transcription of this operon even in the absence of a functional version of RgfD. In the future, additional studies should focus on clarifying the specific regulatory role of *rgfD* during each growth phase and identifying other factors that contribute to transcription. It is important to note that quorum quenching has also been described to occur following entry into stationary phase in several bacterial species [45,46]. Since complementation with the truncated *rgfD* from the WT did not restore *rgfC* expression relative to complementation with a complete *rgfD* from a different strain (genotype), we further hypothesize that *rgfB* may be needed for *rgfD* processing in CC-17 strains containing a truncated RgfD protein. Indeed, the *agrBD* complex was found to be responsible for activation of *agr* in *S. aureus* [42] and hence, future work is required to determine whether extrachromosomal transcription of *rgfBD* from a CC-17 strain can restore *rgfC* activity and if certain mutations within *rgf* can result in altered protein function. Little is known about the structure of the mature RgfD peptide in GBS and virtually nothing is known about how that structure varies across diverse strain types with different *rgfD* alleles.

The *∆rgfD* mutant also had a significant decrease in *rgfC* expression following exposure to decidualized T-HESCs and in its ability to associate with the T-HESCs; the latter could be restored following complementation with a complete *rgfD* from the GB00012 strain. Although the decrease in association was modest and the biological relevance is not clear, it was consistently observed and statistically significant. Because we have previously shown that GBS strains of different genetic backgrounds vary in their ability to attach to A549 lung epithelial cells and T-HESCs [37], it is possible that the *rgf* operon plays a role in regulating distinct adherence factors. These factors may be needed to colonize different tissues inside the host and likely vary across the GBS genotypes. Because *rgf* was previously found to activate *fbsB*, the gene encoding one of two fibrinogen binding proteins [23], it is possible that the reduction in host cell association in the truncated *∆rgfD* CC-17 mutant is due to a decreased ability to bind fibrinogen or other cell components via the lack of *rgf* activation. Similar findings were observed when both *rgfA* and *rgfC* were interrupted in a prior study [23]. Since our prior study demonstrated that only a small fraction (<1%) of associated bacteria invaded host cells [37]; however, the association reduction observed in the GB00451*∆rgfD* mutant cannot be explained solely by *fbsB* activation or through reduced invasion. Support for this hypothesis also comes from the observation that *fbsB* expression was not significantly different in the WT and GB00451*∆rgfD* mutant across the growth phase despite the observed differences in relative transcript levels of *rgfC*. Nonetheless, it is also possible that host cell exposure does not represent the optimal conditions for *rgf* activation given the higher level of *rgfC* expression that we observed during growth at OD_595_ = 0.4. Another possibility affecting host cell association is that the CC-17 strains containing the truncated *rgfD* are less likely to form biofilms due to altered activation of genes important for adherence. Reduced biofilm production has been demonstrated with deletion of the *agr* operon in *S. aureus* [47] and our prior study of biofilms in 293 GBS strains showed that strains belonging to ST-17, which more commonly possessed the truncated *rgfD* in this study, were significantly more likely to form weak biofilms relative to strains from other lineages [33]. Because we also observed an association with weak biofilm production in ST-19 strains, a more comprehensive comparative genomics analysis is warranted. In the present study, most of the ST-19 strains possessed the large deletion within *rgfC* and hence, it is possible that altered transcription of *rgfC* combined with a complete *rgfD* can also impact biofilms. Although there was no association between the *rgfC* deletion and biofilm production overall, it is notable that only one of the nine CC-19 strains formed a strong biofilm and possessed a complete *rgfC*; the remaining eight CC-19 strains had the *rgfC* deletion and formed weak biofilms. Further testing of the different *rgf* mutants is therefore warranted, particularly those clinical strains with natural mutations, which can enhance understanding of the relationship between sequence variation, *rgf* activation and colonization.

The only other verified quorum sensing system in GBS involves RovS, an Rgg-type transcriptional regulator and its activator, a short hydrophobic peptide (SHP), has been found in many specis of *Streptococcus* [48,49]. Similar to the *rgf* system, SHP is post-translationally modified by one or more peptidases and secreted extracellularly [50,51]. Rather than indirectly affecting downstream gene regulation through extracellular recognition, however, the SHP interacts directly with RovS following importation by the Ami oligopeptide transporter [49]. Similar to the *rgf* operon, this system is autoregulating and affects expression of fibrinogen-binding proteins and host-cell attachment [51]; hence, differential expression of the RovS system could have an impact on our findings and warrants further investigation. Interestingly, the SHP has been linked to persistence [51], while inactivation of *rgfC* has specifically been shown to induce a disseminating and invasive phenotype [29,39]. Because the SHP system has also been shown to function differently in different mediums [51], further work should also focus on identifying the optimal conditions for *rgf* expression and *rgf*-associated regulatory networks, particularly during the course of an infection. Additional studies that aim to isolate the mature RgfD peptide from cell-free supernatant cultures and assess its impact on *rgf* expression over time are also needed.

## 5. Conclusions

Because GBS disease progression can involve transcriptional remodeling in response to changing host environments, quorum sensing offers a potential explanation for the variation in pathogenicity that has been observed between strains belonging to different phylogenetic lineages. Although quorum sensing has been demonstrated to affect pathogenesis for many bacterial species, there are few studies specific to GBS. The work described herein adds to the knowledge of quorum sensing systems in GBS and better defines the role of *rgfD*, the gene encoding the putative auto-inducing peptide, as a regulator of *rgfC* and stimulus for host-cell association.

## Figures and Tables

**Table 1:** Strains examined in the study with sequence accession numbers.

Strain	Accession Number	Strain	Accession Number
*rgf* reference sequence	AF390107.1	GB00557	GCA_000290235.1
GB00002	GCA_000289475.1	GB00614	GCA_000290335.1
GB00012	GCA_000288135.1	GB00651	GCA_000290375.1
GB00013	GCA_000288095.1	GB00654	GCA_000290395.1
GB00020	GCA_000288235.1	GB00663	GCA_000290435.1
GB00082	GCA_000288215.1	GB00679	GCA_000290475.1
GB00083	GCA_000288255.1	GB00865	GCA_000290495.1
GB00092	GCA_000290055.1	GB00867	GCA_000289595.1
GB00097	GCA_000289495.1	GB00874	GCA_000289615.1
GB00111	GCA_000290075.1	GB00884	GCA_000289635.1
GB00112	GCA_000291585.1	GB00887	GCA_000289655.1
GB00115	GCA_000290095.1	GB00891	GCA_000290215.1
GB00190	GCA_000290135.1	GB00904	GCA_000288375.1
GB00206	GCA_000289535.1	GB00923	GCA_000288475.1
GB00226	GCA_000288195.1	GB00929	GCA_000288515.1
GB00241	GCA_000288175.1	GB00932	GCA_000288535.1
GB00245	GCA_000288335.1	GB00959	GCA_000288615.1
GB00279	GCA_000288355.1	GB00984	GCA_000288655.1
GB00300	GCA_000289575.1	GB00986	GCA_000289715.1
GB00555	GCA_000290235.1	GB00992	GCA_000289735.1

Accession numbers were assigned by the European Nucleotide Archive (http://www.ebi.ac.uk/ena). Sequences are also available at www.pathogenportal.org/portal/portal/PathPort/Data.

**Table 2:** Oligonucleotide primers used in this study.

Primer/Gene	Forward Primer (5′ to 3′)	Reverse Primer (5′ to 3′)
**Mutagenesis**		
rgfD_del 1 & 2	CCGCGGATCCCCACTTTTACTCATGGGTGACTT	*CCCATCCACTAAACTTAAACA*GCATTCCAAACTTTGTAAGGAGTC
rgfD_del 3 & 4	*TGTTTAAGTTTAGTGGATGGG*TTTTATTCAACAGGCACGTTTAG	GGGGGTACCAAAACTTCTTCAATCCTTCTGCT
rgfD_del 5 & 6	TCATACTCGTCGTGCTCTGG	CAACTCTATGTGACCTTAATGACG
plz12:*rgfD*	CGCGGATCC**AGGAGG**ACAGCTATGCGAAGTTTGGAATGCATGAG	AAAACTGCAGTTCTCTCTAAACGTGCCTGTTG
**qPCR Detection**		
*gyrA*	CGGGACACGTACAGGCTACT	CGATACGAGAAGCTCCCACA
*rgfC*	GCGAAGTAGTGAAGTTTCGCCCAT	CCGGTCTAAACTGGCTATTGCTCC
*rgfB*	GCAAGTACCATGAAGGGGTAGCG	TCAGCTACCAGAGCACGACGAGT
*fbsB*	GCGATTGTGAATAGAATGAGTG	ACAGAAGCGGCGATTTCATT

Underline designates restriction enzyme sites, *Italic* designates complementary sequence, **Bold** designates ribosomal-binding sequence.

## Abbreviations

GBS	Group B *Streptococcus*
qPCR	quantitative polymerase chain reaction
EOD	early onset disease
MLST	multilocus sequence type
STS	signal transduction system
TCS	two component system
ST	sequence type
CC	clonal complex
THB	Todd-Hewitt broth
THG	Todd-Hewitt broth plus 1% glucose
THA	Todd-Hewitt agar
PBS	phosphate-buffered saline
T-HESC	Telomerase-immortalized human endometrial stromal cells
MOI	multiplicity of infection
SNP	single nucleotide polymorphism
